# Editorial: Early Detection and Diagnosis of Cancer

**DOI:** 10.3389/fgene.2022.875421

**Published:** 2022-03-14

**Authors:** Rahul Kumar, Youping Deng, Jian-Bing Fan, Lei Wei

**Affiliations:** ^1^ Computational Genomics and Transcriptomics Laboratory, Department of Biotechnology, Indian Institute of Technology Hyderabad, Telangana, India; ^2^ Department of Quantitative Health Sciences, John A. Burns School of Medicine, University of Hawaii at Manoa, Honolulu, HI, United States; ^3^ AnchorDx Medical Co., Guangzhou, China; ^4^ Department of Biostatistics and Bioinformatics, Roswell Park Comprehensive Cancer Center, Buffalo, NY, United States

**Keywords:** cancer treatment, early detection, diagnosis, survival, prognosis

The efficacy of the cancer treatment largely depends on the stage of its detection. Detecting cancer at an early stage can significantly improve cancer treatment. Unfortunately, early-stage cancer detection is limited by various factors and is still a moonshot. However, recent advancements in the next generation sequencing technologies led to the identification of biomarkers with high prognostic value. This research topic compiled some of the recent research work done in this direction.

Zhu et al. identified five genes (i.e., AP2, CDH1, DACT2, HIN1, and RASSF1A), which were found to be frequently methylated in thyroid cancer. They further used this panel of genes to understand the epigenetic heterogeneity in thyroid cancer and found the association between epigenetic heterogeneity and cancer development and progression.

In a bioinformatics-based analysis, Hu et al. found the association between high expression of PLEC1 and poor prognosis of gastric cancer (GC). They also found SNPs (rs3765524 C > T, rs2274223 A > G and rs3781264 T > C) associated with increased risk of GC. Chemotherapy resistance is a major obstacle in GC patients treated with fluorouracil and cisplatin. Liu et al. identified nine autophagy-related genes (ARGs) signatures associated with the prognosis of chemotherapy patients using TCGA data. They also illustrated the role of increased expression of CXCR4 and its association with survival. Zhang et al. identified a prognostic signature of ten differentially expressed lncRNAs to predict the prognosis of GC. These lncRNAs are related to inflammation and can be explored for the therapeutic opportunity.

Xiao et al. studied the prognostic potential of endoplasmic reticulum (ER) stress-related genes, ATF6, EMC6, XBP1 and CHOP, and apoptosis-related gene, APAF1 in pancreatic cancer (PC). They found that ATF6 upregulation and EMC6 and APAF1 downregulation were significantly associated with poor survival of PC patients.

Wu et al. studied the role of centromere protein N (CENPN) in the pathology of gliomas using clinical and gene expression data from TCGA and CGGA databases. They found the elevated expression of CENPN associated with unfavorable outcomes and further validated their findings in independent clinical specimens. GSEA and ssGSEA analysis revealed the association of CENPN with inflammation, immune-related signaling, and infiltration of immune cells.

Zhao et al. analyzed the pan-cancer expression data and found that secreted phosphoprotein (SPP1) is overexpressed in most of the cancer types including cervical cancer. They also reproduced their findings in GEO database. Based on their analysis, they concluded that SPP1 is significantly overexpressed in cervical cancer than normal cervical epithelial tissues and may be explored as a promising prognostic biomarker for cervical cancer.

Pu et al. carried out a comprehensive bioinformatics analysis of RNA sequencing data and clinical traits of hepatocellular carcinoma (HCC) patients from TCGA. They found six genes, CEP55, DEPDC1, KIF23, CLSPN, MYBL2, and RACGAP1, closely associated with prognosis and immune infiltration. They also proposed the potential of these genes as therapeutic targets or prognostic biomarkers in HCC.

Zheng et al. studied the glycolysis-related lncRNAs using transcriptome and clinical data of bladder cancer (BCa) from TCGA. They identified 59 differentially expressed glycolysis-related lncRNAs, nine (AC099850.3, AL589843.1, MAFG-DT, AC011503.2, NR2F1-AS1, AC078778.1, ZNF667-AS1, MNX1-AS1, and AC105942.1) of which were found to have prognostic significance. These nine lncRNAs can be utilized as biomarkers to distinguish high-risk and low-risk BCa patients.

These nine research studies published under this research topic identified potential biomarkers of high prognostic and diagnostic significance and advanced the field of biomarker development.

